# Association Between Sexual Violence and Reported Anorectal Symptoms Among Adults: A Systematic Review

**DOI:** 10.1007/s00192-025-06423-4

**Published:** 2025-11-22

**Authors:** Tara Reman, Jeanne Bertuit, Veronique Feipel

**Affiliations:** 1 HESAV School of Health Sciences - Vaud, HES-SO University of Applied Sciences and Arts Western Switzerland, 21 Av Beaumont (HESAV), Lausanne, Switzerland; 2 Laboratory of Functional Anatomy, Faculty of Human Movement Sciences, Université Libre de Bruxelles, Brussels, Belgium; 3 Laboratory of Anatomy, Biomechanics and Organogenesis, Faculty of Medicine, Université Libre de Bruxelles, Brussels, Belgium

**Keywords:** Anorectal symptoms, Constipation, Fecal incontinence, Sexual violence systematic review

## Abstract

**Introduction and Hypothesis:**

Individuals experiencing anorectal symptoms may report a history of sexual violence (SV) and conversely, survivors of SV may later report anorectal symptoms. However, the nature of the relationship between SV and these symptoms remains insufficiently understood. This review aims to systematically assess the reported prevalence and severity of anorectal symptoms, as well as their association with sexual violence, among adult survivors of both sexes.

**Material and Methods:**

Following the PRISMA guidelines, a systematic search of three databases, CINAHL, Embase, and MEDLINE/PubMed, was performed for quantitative observational studies concerning SV (rape, sexual harassment, childhood sexual abuse, female genital mutilation) as well as reported anorectal symptoms among adults. Cross-sectional, cohort, case–control, and case series studies published in English and French were included. The Mixed Methods Appraisal Tool (MMAT) was used for assessing the quality of included studies.

**Results:**

Thirteen studies met the inclusion criteria for data extraction. Most studies (12/13) were conducted in a medical setting with a cross-sectional design. Two cross-sectional studies assessed the prevalence of reported anorectal symptoms among SV survivors and 11 studies evaluated the prevalence of SV among patients with anorectal disorders. SV prevalence ranged from 4.1% to 62%. Constipation was the main studied symptom (9/13) followed by fecal incontinence (7/13), anorectal pain (2/13), and recto-vaginal fistula (1/13). Assessments for both SV and anorectal disorders lacked standardization, involving heterogeneous findings.

**Conclusion:**

This review highlights the need for more specific research using standardized assessment of SV and anorectal disorders to better understand their association to improve trauma-informed care pathways.

## Introduction

The World Health Organization (WHO) defines sexual violence (SV) as “any sexual act, attempt to obtain a sexual act, or other act directed against a person’s sexuality using coercion, by any person regardless of their relationship with the victim, in any setting” [1]. This definition includes a broad range of situations such as rape, sexual abuse, forced marriage, denial of the right to use contraceptive equipment or prevention of sexually transmitted diseases, as well as forced abortion and forced prostitution. Men, women, children, and disable people are all found to be victims of SV, and the perpetrators of abuse can range from being someone known to the victim to a complete stranger. Accurately estimating the prevalence of SV is challenging due to underreporting and wide variability across studies. However, a common figure is that roughly 30% of women over the age of 15 have been victims of SV [2]. In Western nations, the prevalence of male-on-male rape or sexual assault is between 5 and 10% of all sexual assaults each year and 6% of children reported experiencing forced sexual intercourse in their lifetime [3, 4]. There are many reasons why victims may not report SV. These can include feelings of shame, fear, or guilt, concerns about possible retaliation from the perpetrator, and not realizing that being forced into sexual acts is a form of SV [5, 6].

SV bears extensive human and financial costs and is considered by some international institutions as a major public health issue worldwide [7]. This has been highlighted in systematic reviews that synthesized estimates on the economic costs of violences, especially against women and children, finding that costs are shouldered by individuals, companies, and governments [8, 9]. At the individual level, there may be medical expenses to treat short- and long-term health consequences of SV and legal costs for those who seek judicial services. Moreover, poor job performance and violence-related absenteeism cost businesses. Finally, violence affects the work of governments that must provide health, social, and judicial services for survivors.

Victimization and sexual abuse have a deep negative impact on survivors’ lives, ranging from general reduced functioning levels and quality of life to lengthened trauma-related symptoms. Survivors often experience serious and lasting impacts on their mental health, including a higher likelihood of developing depression, post-traumatic stress disorder (PTSD), and suicidal ideation [10]. The physical health consequences of sexual assault have been widely studied, with survivors frequently reporting conditions such as injuries, fibromyalgia, diabetes, arthritis, and chronic headaches [11]. Similarly, several systematic reviews have investigated the specific consequences of SV on pelvic health [12–14]. A history of sexual assault is significantly associated with overall gynecological morbidity, especially with pelvic pain, dyspareunia, dysmenorrhea, and urinary incontinence [13]. A recent systematic review demonstrated a significant association between intimate partner violence (which includes SV as well as other forms of abuse) and the risk of functional gastrointestinal disorders (like irritable bowel syndrome or functional dyspepsia) among adult women [14]. However, this review did not evaluate the effects of solely SV on anorectal function, perpetuated by a partner or any type of assaulter. No systematic review to date appears to have specifically explored this link across genders. A better understanding of this relationship could enhance clinical awareness and inform screening strategies in patients presenting with anorectal complaints. This systematic review aims to evaluate the reported prevalence and severity of both SV and anorectal symptoms and to investigate possible associations between the two among adults. We hypothesized that SV survivors would present a higher prevalence as well as more severe reported anorectal symptoms than the general population.

## Materials and Methods

### Protocol and Ethical Approval

The PRISMA guidelines for systematic reviews were used to facilitate the development and reporting of this systematic review [15]. The protocol was reviewed by all the authors and registered with the International Prospective Register of Systematic Review (PROSPERO 2024 CRD42024542070). Ethical approval was not required for this study as it is a systematic review of previously published data. No new data were collected from human participants.

### Information Sources and Search Strategy

A literature search was performed from 8 May 2024 to 01 May 2025 using the following electronic databases: PubMed-MEDLINE, CINAHL, and EMBASE. The search included databases that publish gastroenterology, proctology, and health-related research. Additionally, a manual search of the reference lists of the studies included was conducted to find studies that did not appear in the databases search as per published recommendations and similar topics review. Authors were also contacted via email when there was insufficient data [16]. A combination of similar search terms was used in all databases. The keyword syntax varied according to the database and the requirement of the search strategy. For each database, we combined anorectal symptoms using the OR function in the symptoms string and connected it to the sexual abuse string using the AND function. For PubMed, MeSH and normal text were used to find appropriate studies (Table 1).

**Table 1:** PubMed searching equation

Sexual violence string	("Sex Offenses"[Mesh] OR "Circumcision, Female"[Mesh] OR "Intimate Partner Violence"[Mesh] OR "Domestic Violence"[Mesh:NoExp] OR "Sex Work"[Mesh] OR "Sexual Harassment"[Mesh] OR "Sexual violence*"[tiab] OR "Sexual abuse*"[tiab] OR "Sex offense*"[tiab] OR "Sexual assault*"[tiab] OR "Sexual crime*"[tiab] OR "Rape"[tiab] OR "Rapes"[tiab] OR "Genital mutilation*"[tiab] OR "female circumcision*"[tiab] OR "genital cutting*"[tiab] OR "Spouse Abuse*"[tiab] OR "partner Abuse*"[tiab] OR " Sexual Child Abuse*"[tiab] OR "Forced abortion*"[tiab] OR "Forced prostitution"[tiab] OR "sexual slavery"[tiab] OR "sexual exploitation*"[tiab] OR "human trafficking*"[tiab] OR "sex trafficking*"[tiab] OR "Sexual Harassment*"[tiab])
Anorectal symptoms string	("Rectal Diseases"[Mesh] OR "Fecal Incontinence"[Mesh] OR "Constipation"[Mesh:NoExp] OR "Diarrhea"[Mesh:NoExp] OR "rectal disease*"[tiab] OR "rectal disorder*"[tiab] OR "rectal dysfunction*"[tiab] OR "anorectal disease*"[tiab] OR "anorectal disorder*"[tiab] OR "anorectal dysfunction*"[tiab] OR "Lower Gastrointestinal Functional"[tiab: ~ 1] OR "fecal urgency"[tiab] OR "rectal urgency"[tiab] OR "fecal incontinence"[tiab] OR "anal incontinence"[tiab] OR "flatulence incontinence"[tiab] OR "flatus incontinence"[tiab] OR "passive fecal leakage*"[tiab] OR "increased daytime defecation*"[tiab] OR "nocturnal defecation*"[tiab] OR "tenesmus"[tiab] OR "constipation"[tiab] OR "feeling of incomplete bowel evacuation"[tiab] OR "straining"[tiab] OR "digitation"[tiab] OR "splinting"[tiab] OR "soiling"[tiab] OR "dyssynergic defecation*"[tiab] OR "animus"[tiab] OR "anorectal prolaps*"[tiab] OR "rectal prolaps*"[tiab] OR "anus prolaps*"[tiab] OR "rectal bleeding*"[tiab] OR "anorectal bleeding*"[tiab] OR "vaginal feces"[tiab] OR "rectovaginal fistula*"[tiab] OR "Diarrhea*"[tiab])

### Eligibility Criteria

The inclusion and exclusion criteria were identified on the basis of the Population, Exposure, Comparison, Outcome (PECO) framework (Table 2). Studies were additionally selected if they met the following inclusion criteria: quantitative observational studies, French or English language, and full text of the publication available. No date restriction was applied. Gray literature, non-published works, such as congress abstracts and articles from Clinicaltrial.gov, or other non-peer-reviewed publications were not included. Systematic reviews and meta-analyses were not included in the synthesis, but their reference lists were screened to identify additional eligible primary studies. Adults were defined as individuals aged over 18 years old [17]. The term “anorectal symptoms” was defined according to ICS/IUGA criteria and included: fecal incontinence, anal incontinence, defecatory urgencies, increased frequency of defecation during the day, nocturnal defecation, defecatory straining, tenesmus, incomplete evacuation sensation, decreased or increased rectal sensation, blockage sensation, digitization/splinting, post-defecation leakage, constipation, rectal prolapse, pain during and after defecation, anodyspareunia, anal laxity, rectal bleeding, anal pruritus, flaturia, fecaluria, and the passage of stool and gas through the vagina [18, 19]. Only studies of symptoms reported via questionnaires or clinical interviews were included. Rape (nonconsensual oral/vaginal/anal penetration), sexual harassment, violent acts against sexual integrity (such as female genital mutilation (FGM)), forced prostitution, and forced abortion, whether perpetrated during childhood or adulthood, were included as “sexual violence” in accordance with the WHO definition [1].

**Table 2:** Eligibility criteria

	Inclusion criteria	Exclusion criteria
Population	Human adults (> 18 years old) All gender	Children and teenagers (< 18 years old)
Exposure	Sexual violence as defined by WHO (rape, sexual harassment, genital mutilation, forced abortion, forced prostitution, childhood sexual abuse)	Other types of abuse Sexual violence combined with other types of abuse
Comparator	General population	
Outcome	Functional anorectal symptoms as defined by ICS/IUGA reported by subjects with questionnaires and/or clinical questions	Functional gastrointestinal syndromes such as IBS, dyspepsia Inflammatory bowel disease as Crohn disease Data from clinical exams like defecography and anorectal manometry

*WHO* World Health Organization, *IBS* Irritable Bowel Syndrome, *ICS* International Continence Society, *IUGA* International Urogynecological Association

### Selection and Data Collection Process

The Covidence reference management system (Covidence Systematic Review Software, Veritas Health Innovation, Melbourne, Australia, 2025) was used to organize, screen, select studies, and extract data from the included studies.

All citations identified from initial searching were first imported into Covidence Software, where duplicate citations were removed, after which two reviewers (TRE and JBE) independently screened all article titles and abstracts. For studies meeting initial inclusion criteria, the same two reviewers conducted an independent full text review. Disagreements regarding inclusion or exclusion criteria were resolved by consensus, or through consultation of a senior third reviewer (VFE). The two reviewers conducted independent data extraction using a predetermined data extraction form on Covidence.

### Study Risk of Bias Assessment

All studies meeting inclusion criteria were assessed for quality using the Mixed Method Appraisal Tool (MMAT), a validated and reliable tool that offers criteria specific to each study design [20, 21]. Appraisal was done by two reviewers (XX and XX) independently using the French version of the MMAT. MMAT appraisal criteria for quantitative, nonrandomized studies includes representativeness of the sample, reliable and valid measures of exposure and outcomes, identification of confounding factors and exposure onset. For quantitative descriptive studies, MMAT criteria included sampling strategy, representativeness of the sample, reliable and valid measure of exposure and outcomes, risk of non-response bias and statistical analysis. Results for each MMAT criterion were reported as percentages categorized as “low risk,” “high risk,” or “unclear risk.” A criterion was rated as “low risk” when it was adequately addressed by the study. Conversely, it was rated as “high risk” when the study did not adequately meet the criterion. If the information provided was insufficient or ambiguous, the criterion was rated as having an “unclear risk. In line with MMAT guidance [20, 21], no overall score was calculated. Methodological quality was reported criterion by criterion, indicating for each domain the proportion of studies rated as low, high, or unclear risk. This presentation highlighted specific methodological strengths and weaknesses across studies. None of the studies were excluded on the basis of MMAT scores. The risk of bias for each study was considered in the analysis stage of this systematic review. The certainty of evidence was evaluated descriptively by considering methodological quality (MMAT appraisal), consistency of findings across studies, sample size, and precision of reported outcomes.

### Data Synthesis

A descriptive and narrative synthesis of the included studies was conducted and organized into three thematic parts: (1) prevalence and severity or reported anorectal symptoms among SV survivors; (2) prevalence of SV, severity of anorectal symptoms, and impact on quality of life among patients with anorectal disorders; and (3) relationship between SV, its characteristics, and reported anorectal symptoms. Within each part, consistencies or variations in the magnitude and direction of effects were examined in relation to variables such as type and context of SV, sociodemographic, and medical characteristics of participants (e.g., sex, psychiatric condition), and methodological differences (e.g., study design, measurement tools for anorectal symptoms, and definitions of SV). Because of heterogeneity across studies in terms of population characteristics, prevalence and type of SV, and outcome definitions, results were synthesized narratively and organized according to these sources of variability rather than pooled quantitatively.

## Results

### Study Selection

**Fig. 1:**
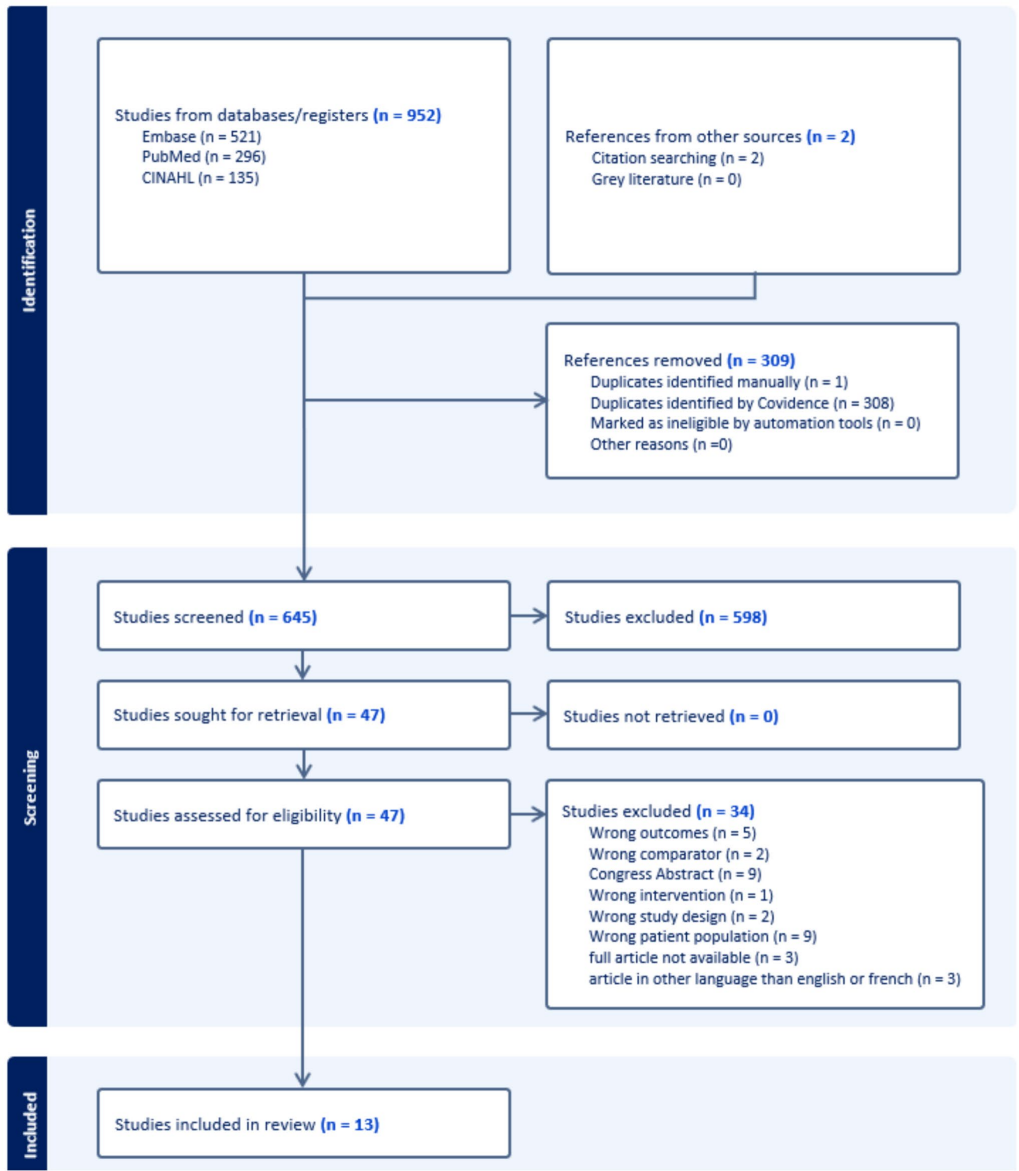
PRISMA flow diagram

The PRISMA flow diagram in Fig. 1 summarizes the study selection. The search protocol identified 954 publications from the online databases, among which 309 were removed because they were duplicate publications. The remaining 645 studies had their title and abstract screened against the eligibility criteria described in Table 2, after which a further 598 were excluded. From the 47 studies selected for full-text review, 34 were excluded due to unsuitable outcomes (*n* = 5), comparator (*n* = 2), intervention (*n* = 1), study design (*n* = 2), population (*n* = 9), being a congress abstract (*n* = 9), full article not being available (*n* = 3), and articles in language other than English or French (*n* = 3). In the end, 13 studies met the inclusion criteria [22–34].

### Study Characteristics

The data included 827 victims of SV among a total of 4868 individuals, most being women (4359 women vs 153 men). Table 3 summarizes the included studies’ characteristics. Most studies were conducted in developed countries (*n* = 12) [22–33], in a medical setting. Regarding design, the majority were cross-sectional studies [23–34] and one was a case series [22]. Two studies explored anorectal symptoms in SV survivors [24, 33] and 11 studies explored SV in patients attending clinics for their anorectal disorders [22, 23, 25–32, 34]. SV was measured using three different approaches: questionnaires [22–24, 26, 27, 30–32], clinical questions [25, 28, 34], and gynecological examination [33]. Most of the studies (*n* = 10) [22, 24, 25, 27–31, 33, 34] examined various characteristics of SV (type of SV (*n* = 5) [22, 24, 27, 29, 33], time of perpetuation of SV (*n* = 5) [24, 27, 29, 30, 34], number of assaulters (*n* = 1) [34], type of penetrative SV (*n* = 6) [22, 25, 27, 28, 31, 34], frequency of SV and type of assaulter (*n* = 2) [27, 29]). The definition of SV differed according to study and included only forced penetrative sex for two studies [28, 31] and FGM for one study [33]. There was also heterogeneity in anorectal symptoms reporting with six studies using unspecified tools [22, 25, 27, 29, 32, 34] and three using unvalidated tools such as self-administrated questionnaires made by the research team [23, 24, 26]. Validated tools included various standardized questionnaires such as Rome II Modular Questionnaire, Wexner Incontinence Scale, Vaizey Incontinence Score, Constipation Scoring System (CSS), Constipation Severity Index (CSI), Obstructed Defecation Score (ODS), and Fecal Incontinence Severity Index (FISI). Constipation was the main symptom studied (*n* = 9) [22, 23, 26–31, 33] followed by fecal incontinence (*n* = 7) [24, 25, 27, 28, 31–33], anorectal pain (*n* = 2) [24, 27], and recto-vaginal fistula (*n* = 1) [34]. Overall, all the studies emphasized the presence/absence of anorectal disorders, but only three studies measured anorectal symptoms severity and the impact of these symptoms on quality of life [28, 31, 33]. Most of the studies (*n* = 9) identified at least one confounding factor for anorectal disorders [23–26, 28, 30–33].

**Table 3:** Included studies characteristics

Authors (year)/country/study design	Sample & settings	Outcomes & assessment	Cofounding factors for anorectal disorders	SV characteristics		
				Definition of SV used	Time of perpetration of SV *n* (%)	Type of penetrative SV *n* (%)
**Pallotta et al. (2014)/Italy/Cross-sectional study **[24]	**Sample:** 67 women victims of abuse aged 18–58 years (median: 33 years, range 28.5–38), 9 victims of sexual abuse **Setting:** 3 Italian anti-violence associations/centers (Florence, Rome, Palermo)	**Ano-rectal symptoms:** Ano-rectal pain, Fecal incontinence Measure: Standardized questionnaire based on the validated Italian version of the Rome II Modular Questionnaire **Sexual Violence:** history of sexual abuse, time of perpetration of sexual abuse Measure: Italian version of the Sexual and Physical Abuse History Questionnaire by Leserman et al.,[53] and Abuse Severity Measure (ASM)	Smoking habit	Unwanted sexual experience included touch, intercourse and other	**Childhood** *n* = 2/9 (22.2%) **Adulthood** *n* = 2/9 (22.2%) **Childhood and Adulthood** *n* = 5/9 (55.6%)	N/A
**Cichowski et al. (2013)/United States of America/Retrospective cross-sectional study **[32]	**Sample:** 1260 women with Pelvic Floor Dysfunction (213 victims of SV, mean age 54.7 ± 15 vs 1048 control, mean age 50.4 ± 12) **Setting:** Urogynecology clinic	**Ano-rectal symptoms:** Fecal incontinence Measure: medical history **Sexual Violence:** history of sexual abuse Measure: interview in person using a standardized, physician-administrated intake questionnaire for SV: Yes/No question	BMI Parity Previous pelvic surgery Ethnicity Psychiatric diagnostic Smoking habit	N/A	N/A	N/A
**Ngongo et al. (2022)/Tanzania, Uganda, Kenya, Malawi, Zambia, Rwanda,** **Ethiopia, Somalia, and South Sudan/Retrospective cross-sectional study **[34]	**Sample:** 26 women with recto-vaginal or recto vesico-vaginal traumatic fistula (15 victims of SV); age N/A **Setting:** Hospital	**Ano-rectal symptoms:** Recto-vaginal fistula and recto-vesico-vaginal fistula Measure: medical history **Sexual violence:** sexual abuse who led to the traumatic fistula Measure: interview in person and information recorded on a standard form	Ethnicity	N/A	**Childhood** *n* = 1/15 (6.6%)	Insertion of foreign body *n* = 2/15 (13.3%)
**Hanna et al. (2023)/Australia/Retrospective cross-sectional study **[31]	**Sample:** 148 female patients with defaecatory dysfunction (25 victims of SV, mean age 48.8 ± 15 vs 123 control, mean age 51.5 ± 17.1) **Setting:** Colorectal Pelvic Floor Unit	**Ano-rectal symptoms:** Fecal incontinence and Constipation Measure: *FI severity:* Wexner Incontinence scale, Vaizey Incontinence Score *FI QoL:* Fecal Incontinence Quality of Life Score *Constipation severity:* Constipation Scoring System, Obstructed Defecation Score *Constipation QoL:* Patient Assessment of Constipation Quality of Life Score **Sexual violence:** history of sexual abuse and type of penetrative SV Measure: Self-administrated questionnaire For SV: 2 unvalidated standardized questions: (1) Have you ever had sex without agreeing to it? (2) If yes, was this vaginal sex, anal sex, or both?	Previous pelvic surgery Episiotomy Perineal tears Psychiatric diagnostic	non-consensual penetrative sex, either vaginal, anal or both	N/A	**vaginal** *n* = 13/25(52%) **anal** *n* = 4/25 (16%) **both** *n* = 8/25 (32%)
**Binkova et al. (2021)/Switzerland/Cross sectional study **[33]	**Sample:** 124 women with FGM (mean age 31.5 ± 7.5) **Setting:** outpatient clinic	**Ano-rectal symptoms:** Fecal Incontinence and Constipation Measure: *FI severity*: Fecal Incontinence Severity *Constipation severity:* Wexner’s constipation scoring system (WCS) *Ano-rectal symptoms on QoL:* Colorectal Anal Impact Questionnaire (CRAIQ-7) **Sexual Violence:** FGM/C and type (I, II, III, infibulation) Measure: gynecological examination	BMI Parity Previous pelvic surgery Episiotomy Perineal tears Ethnicity Smoking habit	any injury to the female genital organs for non-medical reasons	** < 1 year old** *n* = 35/124 (28.2%) **1–5 years old** *n* = 16/124 (12.9%) **6–10 years old** *n* = 40/124 (32.3%) ** > 10 years old** *n* = 11/124 (8.9%) **Unknown** *n* = 22/124 (17.7%)	N/A
**Imhoff et al. (2012)/Unites States of America/Retrospective Cross sectional study **[28]	**Sample:** 1781 women with complaint of fecal incontinence and/or constipation (214 victims of SV, mean age 55.2 ± 13.5 vs 1567 control, mean age 57.1 ± 15.5) **Setting**: tertiary referral center for pelvic floor disorders (Center for Pelvic Physiology)	**Ano-rectal symptoms:** Fecal incontinence and Constipation Measure: *FI severity:* Fecal Incontinence Severity Index *FI QoL:* Fecal Incontinence Quality of Life Scale *Constipation severity:* Constipation Severity Index *Constipation QoL:* Constipation Related Quality of Life **Sexual violence:** history of sexual abuse and type of penetrative SV Measure: Three standardized questions: (1) Have you ever been sexually assaulted or abused? (2) If yes, did it involve vaginal penetration? and (3) If yes, did it involve rectal penetration?	Parity Previous pelvic surgery Ethnicity Psychiatric diagnostic Smoking habit	unwanted sexual experience with a focus on penetrative sex	N/A	**vaginal** *n* = 75/214 (35%) **anal** *n* = 7/214 (3.3%) **both** *n* = 32/214 (14.9%) **declined to define the type** *n* = 100/214 (46.7%)
**O'Brien et al. (2009)/United States of America/Cross sectional study **[25]	**Sample:** 13 patients undergoing subtotal colectomy and iliorectal anastomosis for slow transit constipation (mean age 38.2 ± 7.9), 8 victims of SV (mean age 40.3 ± 8,1). Sex N/A **Setting**: teaching hospital	**Ano-rectal symptoms:** Fecal incontinence Measure: interview **Sexual Violence:** history of sexual abuse, type of penetrative SV Measure: Patients asked two times (no information for the first time) for the 2nd time: interview in person, gentle questions	Psychiatric diagnostic	N/A	N/A	**vaginal** *n* = 2/8 (25%) **anal** *n* = 0/8 (0%) **both** *n* = 6/8 (75%)
**Solé et al. (2009)/Argentina/Retrospective case series **[22]	**Sample:** 121 patients evaluated for obstructed defecation (17 men/104 women, mean age 53 ± 15); 5 victims of SV **Setting:** general gastroenterology practice	**Ano-rectal symptoms:** Obstructed defecation Measure: N/A **Sexual violence:** history of sexual abuse: attempts, forced sexual touching, rape and type of penetrative SV Measure: Sexual and Physical Abuse History Questionnaire by Leserman et al., [53]	N/A	unwanted sexual experience included touch, intercourse and other	N/A	N/A
**McCrea et al. (2009)/United States of America/Retrospective Cross sectional study **[26]	**Sample:** 518 patients with primary diagnosis of constipation (409 women/109 men, median age 51.5 years (range N/A)); 59 victims of SV (56 women/3 men) **Setting:** Center for Pelvic Physiology	**Ano rectal symptoms:** Constipation (stool frequency, anal blockage, pain, need to strain, digital disompaction, feeling of incomplete evacuation) Measure: unvalidated self-administered questionnaire **Sexual violence** history of sexual abuse Measure: unvalidated self-administered questionnaire	Previous pelvic surgery Ethnicity Psychiatric diagnostic	N/A	N/A	N/A
**Rao et al. (2004)/United States of America/Cross sectional study **[23]	**Sample:** 118 patients with constipation (27 men/91 women, mean age 49 years, range 18–85 years); 26 victims of SV **Setting:** tertiary care center	**Ano-rectal symptoms:** Constipation (stool frequency, need to strain, digital disompaction, feeling of incomplete evacuation, urge for defecation) Measure: unvalidated self-administered questionnaire **Sexual violence:** history of sexual abuse Measure: unvalidated self-administered questionnaire	N/A	N/A	N/A	N/A
**Hobbis et al. (2002)/United Kingdom/Cross sectional study **[30]	**Sample:** 207 female patients and subjects (53 CIC, 50 IBS, 51 Crohn, 53 control), mean age 38.9 ± 12.9; Number of victims of SV N/A **Setting:** gastroenterology department	**Ano-rectal symptoms:** Constipation Measure: Bowel disease questionnaire [54] **Sexual Violence:** history of sexual abuse and period Measure: Sexual and Physical Abuse History Questionnaire by Leserman et al. [53]	Ethnicity	any reported unwanted sexually oriented experience from exposure of the sex organs to non-consenting intercourse	**Childhood** CIC *n* = 12/53 (22.6%) Control *n* = 9/53 (17.0%) *p* > 0.05 **Adulthood** CIC *n* = 18/53 (34.0%) Control *n* = 12/53 (22.6%) *p* < 0.05	N/A
**Leroi et al. (1995)/Canada, France (1)/Cross sectional study **[29]	**Sample:** 91 women, 40 victims of SV with gastroenterological complains (mean age 46.7 ± 1.9) **Setting**: outpatient clinic	**Ano-rectal symptoms** Straining at stool, constipation, digital evacuation of the rectum Measure: interview, medical history **Sexual violence:** history of sexual abuse, periode of abuse, fréquence of sexual abuse, nature of sexual abuse, abuser Measure: N/A	Previous pelvic surgery	any action of a sexual nature done intentionally, by the abuser, against the will of the victim. Included exhibitionism, touching, penetration into the vagina, anus or mouth	**Childhood** 10/40 (25%)	**Anal** 6/40 (15%)
**Leroi et al. (1995)/Canada, France (2) Cross sectional study **[27]	**Sample**: 344 patients, age and sex N/A (89 victims of SV, age and sex N/A) **Setting:** (A) laboratory (B) specialized tertiary care universtiy hospital, (C) gastroenterologist in private practice	**Ano-rectal symptoms:** constipation, fecal incontinence, rectal pain Measure: patient complaint **Sexual violence:** exhibitionism, touching, rape and type of penetrative SV, age during SV, abuser, SV frequency Measure: Interview in person with questionnaires	N/A	any action of a sexual nature done intentionally, by the abuser, against the will of the victim. Included exhibitionism, touching, penetration into the vagina, anus or mouth	**Childhood** GA *n* = 24/89 (27%) before 19 years old GB N/A GC N/A	GA **full coitus** *n* = 11/89 (12.4%) **oral sex** *n* = 3/89 (3.4%) **anal sex** *n* = 6/89 (6.7%) GB N/A GC N/A

*N/A* Not applicable, *SV* Sexual violence, *BMI* Body mass index, *FI* Fecal incontinence, *QoL* Quality of life, *FGM* Female genital mutilation, *CIC* Chronic idiopathic constipation, *IBS* Irritable bowel syndrome, *GA* Group A, *GB* Group B, *GC* Group C

Meta-analysis was not used for this review as selected studies were considered too heterogeneous in terms of measurement of the outcomes.

### Risk of Bias in Studies

**Fig. 2:**
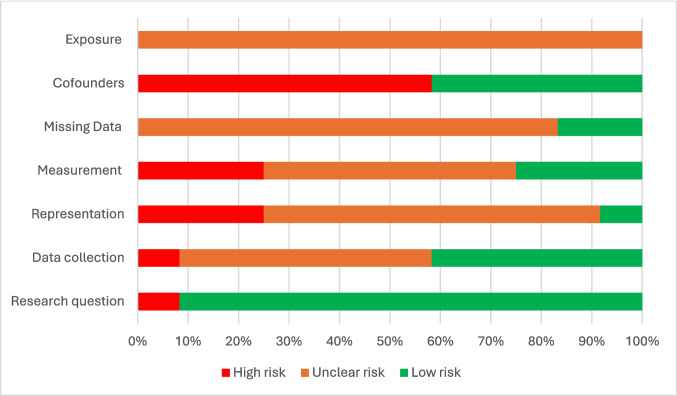
Assessment of quality of nonrandomized included studies using the MMAT tool [20, 22–32]

Using the MMAT assessment tool, none of the quantitative descriptive studies were deemed at “low risk” of bias in all five MMAT categories, and eight studies had at least one item deemed at “high risk” [23–25, 27, 29–31, 34]. More specifically, management of missing data and representativeness of the target population were deemed at low risk for only two studies [28, 30]. For seven studies, confounders were not accounted for in the analysis of the studies [23, 25, 27, 29–31, 34] (Fig. 2). The quality appraisal of the single descriptive study [22] showed a clear research question, adequate data collection, and clear presentation (all rated low risk). However, the sampling strategy, measurement, nonresponse bias, and statistical analysis were rated as unclear risk. Overall, the included studies were rated as having a moderate to high risk of bias.

### Narrative Synthesis of Results

#### Prevalence and Severity of Reported Anorectal Symptoms Among SV Survivors

Two studies investigated the prevalence and severity of anorectal symptoms among survivors of SV [24, 33] (Table 4). Pallotta et al., in a study including 67 women with a history of sexual abuse, reported a prevalence of 17.9% for anorectal pain and 3% for fecal incontinence in women who had suffered sexual abuse [24]. Binkova et al. studied 124 women who had undergone FGM and found a prevalence of 10.5% for constipation. The severity of the fecal incontinence and constipation symptoms found in this population was generally mild (FISI 2.1 ± 6.3; WCS 7.4 ± 5.9) and lower than that found in the general female population suffering from the same dysfunctions [33].

**Table 4:** Prevalence and severity of reported anorectal symptoms among SV survivors

Author(s) (year)	Prevalence of anorectal disorders *n*(%)	Severity of anorectal disorders mean ± SD	Control group mean ± SD
**Pallotta et al. (2014) **[24]	ano rectal pain *n* = 12 (17.9%) fecal incontinence *n* = 2 (3.0%)	N/A	N/A
**Binkova et al. (2021) **[33]	fecal incontinence N/A constipation *n* = 13 (10.5%)	FISI 2.1 ± 6.3 WCS 7.4 ± 5.9	*Fecal Incontinence* Uncut women with FI 38.6 ± 10.7 General population in Netherland 23.2 ± 15.0 *Constipation* Women with PFD > 15

*N/A* Not applicable, *FISI* Fecal Incontinence Severity Index, *WCS* Wexner Constipation Score, *SD* Standard deviation, *FI* Fecal incontinence, *PFD* Pelvic floor dysfunction

#### Prevalence of SV, Anorectal Symptoms Severity and Impact on Quality of life Among Patients Attending Clinics for Anorectal Disorders

Nine studies assessed the prevalence of SV among patients presenting to medical consultations with an anorectal complaint [22, 23, 25–28, 31, 32, 34]. Reported prevalence ranged from 4.1% to 62%, with the lowest prevalence observed among 121 patients suffering from obstructed defecation (4.1%) [22] and the highest among 13 patients who had undergone colorectal surgery for constipation (62%) [25] and 26 patients with traumatic recto-vaginal or recto-vesico-vaginal fistulas (42.3%) [34], as described in Table 5.

**Table 5:** Prevalence of reported SV and anorectal symptoms among patients with anorectal complaints

Author(s) (year)	Prevalence reported SV *n* (%)	Prevalence of anorectal symptoms *n* (%)
**Cichowski et al. (2013) **[32]	*n* = 33 (23.1%)	*fecal incontinence* Control *n* = 110 (10.5%) SV *n* = 33 (15.5%) *p* = 0.037
**Ngongo et al. (2022) **[34]	*n* = 11 (42.3%)	*Recto-vaginal fistula* Control *n* = 13 (21.3%) SV *n* = 9 (60%) *Vesico-recto-vaginal fistula* Control *n* = 2 (3.3%) SV *n* = 2 (13%)
**Hanna et al. (2023) **[31]	*n* = 25 (17.0%)	N/A
**Imhoff et al. (2012) **[28]	*n* = 214 (12.0%)	N/A
**O'Brien et al. (2009) **[25]	*n* = 8 (62%)	N/A
**Solé et al. (2009) **[22]	*n* = 5 (4.1%)	N/A
**McCrea et al. (2009) **[26]	*n* = 59 (11.4%)	N/A
**Rao et al. (2004) **[23]	*n* = 26 (22%)	Stool frequency *p* > 0.11 Need to strain *p* > 0.11 Digital disimpaction *p* > 0.11 feeling of incomplete evacuation *p* < 0.01 urge for defecation *p* < 0.01
**Hobbis et al. (2002) **[30]	N/A	N/A
**Leroi et al. (1995)** [29]	N/A	*Constipation* VS *n* = 30/40 (75%) Control *n* = 21/31 (67.7%) *p* > 0.05 *Straining at stool* VS *n* = 21/40 (52.5%) Control *n* = 21/31 (67,7%) *p* > 0.05 *Digital evacuation of the rectum* VS *n* = 4/40 (10%) Control *n* = 3/31 (10%)
**Leroi et al. (1995) **[27]	*n* = 106 (53,8%) *n*GA = 27 (34%) *n*GB = 60 (42%) *n*GC = 19 (40%)	GA (*n* = 79) *constipation* VS *n* = 24 (88%) Control *n* = 30 (57%) *p* < 0.05 *Rectal pain* VS *n* = 11 (42%) Control *n* = 27 (51%) *p* > 0.05 *fecal incontinence* VS *n* = 6 (23%) Control *n* = 11 (21%) *p* > 0.05

*SV* Sexual violence, *N/A* Not applicable, *GA* Group A, *GB* Group B, *GC* Group C

Five studies investigated the difference in prevalence of anorectal disorders between survivors of SV and patients not exposed to this type of violence [23, 27, 29, 32, 34]. Among these clinical studies, three found a significant difference between patients with and without a history of SV: Cichowski et al., in a cohort of 1260 patients with pelvic floor dysfunction reported a higher prevalence of fecal incontinence in SV survivors (15.5%) compared with non-SV patients (10.5%) [32]; Rao et al., in a sample of 118 patients with constipation, observed a higher rate of sensation of incomplete rectal emptying, and defecatory urgency among SV survivors [23]; and Leroi et al., in 344 patients with gastrointestinal symptoms, reported higher rates of constipation (88% in SV vs. 57% in non-SV) [27].

The severity and impact on quality of life of fecal incontinence and constipation found in female patients survivors of SV were compared to a group of patients not exposed to this type of violence in two studies [28, 31]. The first study by Imhoff et al. in 1781 patients with fecal incontinence or constipation, investigated this issue using the FISI and CSI for severity, and Faecal Incontinence Quality of Life Score (FIQL) and Constipation Related Quality of Life (CR-QOL) for impact on quality of life. For the two anorectal disorders studied, the authors found a significant difference between the groups with a greater severity in the group of surviving patients. The impact of these anorectal symptoms on quality of life was also significantly larger than in the control group [28]. A more recent study by Hanna et al., in 148 patients with defaecatory dysfunction, used a similar methodology to assess the same criteria but with different measuring instruments (Wexner Incontinence Scale, Vaizey Incontinence Score, CSS, and ODS for severity, and FIQL and Patient Assessment of Constipation Quality of Life Score (PACQL) for impact on quality of life). The authors did not find a significant difference between the groups for all evaluated criteria (severity and impact on quality of life for both symptoms) (Table 6) [31].

**Table 6:** Constipation and fecal incontinence severity and impact on quality of life among survivors’ patients compared to control group

Authors	Constipation						Fecal incontinence					
	Symptom severity			Impact on quality of life			Symptom severity			Impact on quality of life		
	Score SV group (mean (SD))	Score control group (mean (SD))	*P* value	Score SV group (mean (SD))	Score control group (mean (SD))	*P* value	Score SV group (mean (SD))	Score control group (mean (SD))	*P* value	Score SV group (mean (SD))	Score control group (mean (SD))	*P* value
**Hanna et al. (2023) **[31]	CSS 15.9 (7.1) ODS 13.9 (6.7)	CSS 12.9 (5.4) ODS 13.2 (6.1)	CSS *p* = 0.10 ODS *p* = 0.70	PACQL—Dissatisfaction 77.6 (30.4) PACQL—Satisfaction 8.77 (4.6)	PACQL—Dissatisfaction 67.6 (25.6) PACQL—Satisfaction 8.45 (4.2)	PACQL—Dissatisfaction *p* = 0.22 PACQL—Satisfaction *p* = 0.81	Wexner 10.4 (5.4) Vaizey 12.9 (5.0)	Wexner 10.1 (4.3) Vaizey 12.3 (5.5)	Wexner *p* = 0.83 Vaizey *p* = 0.73	FIQL total 9.48 (2.9) FIQL lifestyle subscale 2.86 (0.9) FIQL coping/behavior subscale 1.91 (0.7) FIQL depression/self-perception subscale 2.57 (0.9) FIQL embarrassment 2.15 (0.7)	FIQL total 10.3 (3.3) FIQL lifestyle subscale 2.91 (0.9) FIQL coping/behavior subscale 2.19 (0.9) FIQL depression/self-perception subscale 2.87 (0.9) FIQL embarrassment 2.37 (1.0)	FIQL total *p* = 0.36 FIQL lifestyle subscale *p* = 0.83 FIQL coping/behavior subscale *p* = 0.27 FIQL depression/self-perception subscale *p* = 0.25 FIQL embarrassment *p* = 0.44
**Imhoff et al. (2012) **[28]	CSI total 42.1 (14.3) CSI OD 20.7 (6.3)	CSI total 36.0 (15.2) CSI OD 18.8 (6.4)	CSI total *p* < 0.001 CSI OD *p* = 0.007	CR-QOL total 57.6 (18.7) CR-QOL eating subscale 9.9 (4.3) CR-QOL bathroom subscale 12.9 (5.2) CR-QOL social impairment subscale 12.1 (6.7) CR-QOL distress subscale 24.5 (4.9)	CR-QOL total 50.9 (17.7) CR-QOL eating subscale 8.6 (4.1) CR-QOL bathroom subscale 10.9 (5.5) CR-QOL social impairment subscale 9.5 (5.8) CR-QOL distress subscale 22.8 (7.0)	CR-QOL total *p* = 0.009 CR-QOL eating subscale *p* = 0.01 CR-QOL bathroom subscale *p* = 0.004 CR-QOL social impairment subscale *p* = 0.001 CR-QOL distress subscale *p* = 0.19	FISI 33.2 (13.6)	FISI 29.9 (13.2)	*p* = 0.002	FIQL total 7.41 (3.3) FIQL lifestyle subscale 2.18 (1.0) FIQL coping/behavior subscale 1.75 (0.8) FIQL depression/self-perception subscale 2.02 (0.9) FIQL embarrassment subscale 1.87 (0.8)	FIQL total 8.43 (4.0) FIQL lifestyle subscale 2.57 (1.1) FIQL coping/behavior subscale 1.99 (0.9) FIQL depression/self-perception subscale 2.40 (1.1) FIQL embarrassment subscale 2.03 (0.9)	FIQL total *p* = 0.002 FIQL lifestyle subscale *p* < 0.001 FIQL coping/behavior subscale *p* < 0.01 FIQL depression/self-perception subscale *p* < 0.01 FIQL embarrassment subscale *p* < 0.01

*CSS* Constipation scoring system, *ODS* Obstructed Defecation Score, *CSI* Constipation Severity Index, *CSI OD* Constipation Severity Index Obstructed Defecation subscale, *PACQL* Patient Assessment of Constipation Quality of Life Score, *CR-QOL* Constipation Related Quality of Life, Wexner Wexner Incontinence scale, Vaizey Vaizey Incontinence Score, *FISI* Fecal Incontinence Severity Index, *FIQL* Faecal Incontinence Quality of Life Score, *SD* Standard deviation

#### Relationship Between SV, its Characteristics and Reported Anorectal Symptoms

Five studies evaluated the association between SV and the presence of anorectal symptoms [24, 26, 28, 32, 33]. Only the study by Imhoff et al. highlighted a significant association between the severity of fecal incontinence symptoms, their impact on quality of life, and the presence of a history of SV among patients suffering from anorectal disorders, controlling for confounding factors (age, ethnicity, psychiatric condition, episiotomy, hysterectomy) [28]. The severity of constipation symptoms was also significantly associated with the presence of a history of SV when controlling for confounding factors (age, ethnicity, psychiatric condition, episiotomy). Only Pallotta et al. evaluated the association between the time of perpetration of the abuse and the number of reported anorectal symptoms. They did not find an association between these two variables and likewise, they found no significant association between the presence of SV alone and the number of anorectal symptoms [24]. Finally, Binkova et al. evaluated the association between type of FGM, age at FGM, past violent events like sexual abuse and the impact of pelvic floor symptoms on quality of life using PFIQ-7 scores (including colorectal–anal impact score) through a multivariable model. Past violent events and age at FGM of more than 10 were significantly associated with a higher impact of pelvic floor symptoms on quality of life among women with FGM [33] (Table 7).

**Table 7:** Association between SV and anorectal symptoms

Authors	Aim	Multivariate model	Findings
**Pallotta et al. (2014) **[24]	To assess the association between number of reported GI symptoms (including rectal pain and fecal incontinence) and variables as time of perpetration of the abuse, its type and socio-demographic characteristics	Age Level of Education Economic self sufficiency BMI Smoking habit Alcohol consumption Time of perpetration of abuse Type of abuse Severity of abuse	Association between the presence of physical plus sexual abuse and the number of GI symptoms No association between the time of perpetration of the abuse (childhood vs. adulthood vs childhood and adulthood) and the number of reported GI symptoms No significant association between the number of GI symptoms and only one type of abuse (sexual or physical) No association between age, economic self-sufficiency, BMI, smoking, alcohol, self-perceived stress and the number of reported GI symptoms
**Cichowski et al. (2013) **[32]	To identify whether a sexual abuse history was associated with PFD (including fecal incontinence)	Age Anxiety No partner Chronic pelvic pain Fecal incontinence	No significative association between fecal incontinence and a history of sexual abuse
**Imhoff et al. (2012) **[28]	To evaluate the independent effect of SV on the responses to disease-specific surveys (FISI, FIQL, CSI, and CR-QOL)	Fecal incontinence severity and impact on QoL Age Ethnicity psychiatric illness episiotomy hysterectomy Constipation, severity and impact on QoL Age Ethnicity psychiatric illness episiotomy	Association between increased severity of fecal incontinence and a history of sexual violence Association between increased impact on quality of life of fecal incontinence and a history of sexual violence Association between increased severity of constipation and a history of sexual violence No significative association between an increased impact on quality of life of constipation symptoms and a history of sexual violence
**McCrea et al. (2009) **[26]	To determine whether gender and the occurrence of various comorbid conditions were associated with an increase in the frequency of constipation-associated characteristics, symptoms, and bowel and dietary habits	Gender (female) Hemorrhoids Depression IBS Chronic Back Pain Arthritis History of SV	No significative associations between a history of sexual assault and any of the characteristics of constipation, its symptoms, and bowel and dietary habits
**Binkova et al. (2021) **[33]	To assess the association between possible risk factors and higher scores for impact of pelvic floor symptoms (including anorectal symptoms) in women with FGM	Type of FGM Infibulation Age at FGM Past violent events Time in Switzerland Age BMI Ongoing pregnancy Parity Previous episiotomy	Past violent events (like sexual abuse) and age at FGM of more than 10 are significantly associated with higher PFIQ-7 scores

*PFIQ-7* Pelvic Floor Impact Questionnaire including ColoRectal-Anal Impact Questionnaire (CRAIQ-7), *BMI* Body mass index, *GI* Gastro-intestinal, *PFD* Pelvic floor dysfunction, *FISI* Fecal Incontinence Severity Index, *FIQL* Faecal Incontinence Quality of Life Score, *CSI* Constipation Severity Index, *CR-QOL* Constipation Related Quality of Life, *QoL* Quality of life, *SV* Sexual violence, *FGM* Female genital mutilation

Across the included studies, findings were heterogeneous, with some reporting significant associations between SV and anorectal symptoms while others did not. This heterogeneity is taken into account in the following “Discussion and Conclusion,” although greater weight was given to studies with larger sample sizes when formulating the overall interpretation [28, 32].

## Discussion

This systematic review presents an analysis of 13 studies that explore the relationship between reported anorectal symptoms and SV. Although the overall findings are heterogeneous, the prevalence of certain anorectal symptoms such as constipation and fecal incontinence appears at a higher rate among patients who have experienced sexual abuse, compared with patients without SV, depending on the type of SV. When considering the general population, constipation is estimated to affect approximately 2.4 to 39.6% of adults and fecal incontinence around 7.7% of community-dwelling adults [35, 36]. The prevalence observed among SV survivors in this review often falls within the higher end of these ranges, although direct comparison remains limited as population-based studies typically do not assess or adjust for SV exposure. The severity of these symptoms, as well as the impact on quality of life, seems to be greater in this population, particularly among patients suffering from fecal incontinence.

Variability in the prevalence of SV among patients reporting anorectal symptoms was found across the included studies. This heterogeneity can be partly explained by the different definitions of SV used. While eight of the 13 included studies provided a definition of SV [22, 24, 27–31, 33], a quarter focused solely on penetrative sexual assaults [28, 31]. This definition significantly narrows the scope of possible forms of SV and limits the identification of survivors of non-contact SV, such as sexual harassment or exhibitionism. Moreover, owing to rape culture and the myth of the “real” SV victim, many survivors do not identify or relate to certain narrow definitions of SV, particularly those who have experienced SV from an intimate partner or family member [37, 38]. SV is also a social taboo subject—few victims dare to speak about it with close relations or with healthcare professionals, even when asked [39, 40]. Therefore, building a trusting therapeutic relationship and creating a long-term safe environment is necessary to allow SV survivors to disclose such sensitive information. This is illustrated by O’Brien’s study, which found a higher prevalence of SV in their sample when patients were asked again after undergoing colectomy surgery [25]. The sensitive nature of the subject makes it difficult to standardize SV assessment methods, which impacts the reported prevalence. Only three of the included studies used the same validated questionnaire [22, 24, 30]. Among them, Hobbis et al., chose to add a more personalized approach through clinical interviews to respect the sensitivity of each individual case [30]. Finally, most of the included studies were conducted in hospital settings, so the reported prevalence of SV applies to patients with anorectal disorders rather than the general population. Selection bias must be considered, especially due to the underrepresentation of certain minorities who face barriers to healthcare access, such as the LGBTQIA+ community or non-native-speaking migrants—populations that are also at higher risk of experiencing SV [41, 42]. The wide range in the reported prevalence of SV across studies—from 4.1% [22] to 62% [25]—likely does not reflect the true prevalence of SV among patients presenting with anorectal disorders. Nonetheless, these figures are broadly consistent with prevalence estimates observed in other populations without anorectal symptoms.

Although this review considered both female and male survivors of SV, where five of the included studies included participants of both genders [22, 23, 25–27], none of these studies evaluated the impact of gender on the severity or prevalence of anorectal symptoms. Only McCrea et al. included gender in their logistic regression model to assess its association with constipation symptoms, but it was evaluated independently of the SV variable [26]. While none of the considered papers included gender along with SV in their modeling, research suggests that gender significantly influences the prevalence and characteristics of anorectal disorders. Fecal incontinence, for instance, is more common in women than in men [43]. Women also tend to experience more severe constipation symptoms and report a greater impact on quality of life compared to men [44]. Therefore, gender could potentially influence the findings and contribute to the heterogeneity observed in this review.

As with the inconsistent definitions of SV, there was considerable variability in the definitions and measurement tools used to assess anorectal symptoms across the included studies. Only two studies employed comparable questionnaires to evaluate the impact of fecal incontinence on quality of life [28, 31]. Symptoms related to constipation, as defined by the Rome IV criteria, also varied between studies [23, 26, 29]. This methodological variability complicates comparisons between studies and limits the generalizability of the findings.

The effect of specific characteristics of the SV, such as age at the time of the assault, on the anorectal symptoms experienced by patients was explored in several studies [24, 29, 33]. Existing literature suggests that early and repeated exposure to interpersonal violence, particularly SV during childhood, increases the risk of developing complex PTSD, which in turn heightens the likelihood of severe somatization, including significant impact on gastrointestinal health [45, 46]. However, Pallotta et al. did not find an association between childhood abuse and the number of reported gastrointestinal symptoms [24]. This finding aligns with the study by Leserman et al., which examined the impact of age at the time of first sexual and/or physical abuse in a sample of 239 female patients attending a gastroenterology clinic. The study found no significant difference in health outcomes between those abused in childhood and those abused in adulthood [47]. In contrast, the study by Binkova et al. highlighted the significant influence of age at the time of FGM on the presence and distress to pelvic symptoms. Girls who underwent FGM after the age of 10 had clearer recollections of the trauma compared to those cut in early childhood or infancy. In cases of this specific form of SV, undergoing FGM after age 10 was significantly associated with a greater impact on pelvic symptoms, including anorectal symptoms [33].

Penetrative forms of SV (anal and vaginal) were reported in six of the included studies [25, 27–29, 31, 34]. However, none of them assessed the impact of these specific types of SV on the prevalence or severity of anorectal symptoms. Only the study by Leroi et al. compared anal manometry data between survivors of vaginal and anal rape but found no significant differences [29].

Two studies provided information on the frequency of SV exposure (e.g., single vs. repeated episodes), but these data were not incorporated into the authors’ analyses of anorectal outcomes [27, 29]. Some of the included studies identified other forms of violence and trauma associated with SV as well, such as physical violence [22, 24, 27, 30]. In the study by Pallotta, physical violence was included in multivariate analyses, which revealed a significant association between the combination of physical and sexual abuse and the number of gastrointestinal symptoms [24]. This finding aligns with existing research indicating that polyvictimization—the experience of multiple forms of violence—is associated with worse health outcomes among survivors of SV [48, 49]. Women exposed to multiple types of violence report higher rates of poor self-rated health, chronic medical conditions, depression, and sexual dysfunction [50]. The multiplicity of violent acts, or polyvictimization, falls within the framework of the continuum of violence [51] which posits that survivors of SV rarely experience a single form of violence but rather multiple forms throughout their lives. This concept is particularly illustrated in the study by Binkova, where one out of two women in the sample reported past violent events beyond FGM or forced/arranged marriage. These past violent experiences were significantly associated with higher levels of distress and a greater impact of pelvic floor symptoms, including anorectal symptoms [33].

Although this review did not specifically examine the mechanisms underlying the relationship between SV and anorectal symptoms, several of the included studies explored potential pathophysiological explanations for this association. Four studies assessed the anorectal physiology of participants to determine whether differences in symptom severity—primarily constipation and fecal incontinence—could be attributed to altered anal canal function [27–29, 31].

The two studies by Leroi et al. reported that women with a history of SV exhibited alterations in anal physiology, as measured by manometry, rectometrogram, and electromyography (EMG) [27, 29]. Specifically, they observed increased pressure in the lower anal canal during defecatory strain, which may result from asynchronous contraction of the external anal sphincter. The authors hypothesized that, following the trauma of sexual abuse, any sensation of rectal fullness may trigger traumatic memories and provoke involuntary contraction of the pelvic floor muscles, leading to stool retention. This abnormality, referred to by the authors as anismus or anorectal dyssynergia, was not identified in the studies by Imhoff et al. and Hanna et al. [28, 31].

Both of these studies employed similar methodologies, using a threshold of a 25% increase in anal canal pressure during straining to define dyssynergia, complemented by a balloon expulsion test. However, unlike the findings reported by Leroi et al.[29], Imhoff and Hanna found no significant differences in anorectal physiological parameters between SV survivors and control participants across all manometric outcomes [28, 31]. According to these authors, the exacerbation of anorectal symptoms in individuals with a history of sexual abuse cannot be explained by dysfunction of the anal sphincter. Similarly, Hanna et al. did not identify a higher prevalence of anal sphincter defects on endoanal ultrasound among SV survivors that could account for the increased severity of fecal incontinence symptoms in this population [31].

The heightened perception of anorectal symptoms among survivors of SV may be linked to broader components of the biopsychosocial model, particularly mental health. This is highlighted in the systematic review by Banjar et al., which demonstrates that psychological factors—such as depression, anxiety, and PTSD—play a particularly important role in moderating symptom severity and persistence. This influences care-seeking behavior and affects treatment outcomes, particularly in cases of functional gastrointestinal disorders like irritable bowel syndrome (IBS) among women who have experienced intimate partner violence [14]. In this review, five of the included studies assessed the prevalence of psychiatric disorders within their samples [25, 26, 28, 31, 32]. Two of them reported significantly higher rates of psychiatric conditions—specifically anxiety and depression—among SV survivors and a significant association between these psychological issues and the presence of pelvic floor disorders, including anorectal symptoms [28, 32]. The involvement of these multiple psychological mechanisms is also supported by pathophysiological explanations for pelvic symptoms in SV survivors, such as lower urinary tract symptoms (LUTS), as demonstrated in the systematic review by Selai et al. [12].

This systematic review is the first to synthesize existing literature on the association between SV and anorectal symptoms among adults, addressing a clinically relevant but underexplored topic. By capturing a wide range of anorectal symptoms and forms of SV, it offers a nuanced understanding of how these experiences may interact. Compared with previous reviews focusing on urinary and gynecological outcomes [12, 13] or functional gastrointestinal disorders in women exposed to intimate partner violence [14], this review specifically addresses anorectal symptoms, in relation to SV, includes both sexes and diverse contexts of SV and examines prevalence, severity, and quality-of-life impact. Despite the application of rigorous methodology, this review has several limitations. First, the search may not have captured all relevant literature, as it was restricted to studies published in English and French and excluded gray literature, dissertations, and theses. Second, this review is based on a limited number of studies (*n* = 13), the majority of which were cross-sectional. This restricts the ability to draw causal inferences between SV and anorectal disorders [23–34]. Third, there was considerable heterogeneity in how both SV and anorectal symptoms were assessed. Over 25% of the quantitative studies did not employ measures with established reliability and validity, which may compromise data quality and reduce the sensitivity to detect true associations [25, 27, 29]. The absence of a standardized definition of SV contributed to variability in study populations, and many studies lacked standardized assessments of key characteristics, including type, frequency, timing, and perpetrator identity, as well as the developmental stage at which the abuse occurred. While this review included studies with participants of all genders, none of the included studies analyzed the potential moderating effect of gender on symptom severity or prevalence. Overall, the certainty of the evidence was judged to be low, given the predominance of cross-sectional designs, small sample sizes, heterogeneity in definitions of SV and anorectal symptoms, and inconsistency of findings across studies. Given these methodological inconsistencies, a meta-analysis could not be performed. Although the findings of this review suggest a potential association between SV and increased severity of anorectal symptoms, both significant and nonsignificant results were observed across studies. Further high-quality, longitudinal research is needed to clarify the nature and mechanisms of this relationship.

## Conclusion

This review shows that the prevalence of SV among patients with anorectal disorders is variable but consistently non-negligible. Greater awareness among healthcare providers of the association between SV and these symptoms is essential for delivering optimal care. Systematic screening for SV—using a broad definition such as “unwanted sexually oriented experience”—should be implemented to identify potential survivors and facilitate appropriate referral to judicial, social, and psychological support services. It is also important to document certain characteristics of the SV experience to assess polyvictimization, its psychological impact, and the severity of somatization. The principles of trauma-informed care—including safety, trustworthiness, transparency, choice, collaboration, and empowerment—should guide clinical examinations and treatment to minimize the risk of retraumatization by healthcare providers [52]. Although findings across studies are heterogeneous, larger studies tend to indicate that survivors of SV present with more frequent and more severe anorectal symptoms compared with patients who have not been exposed to such violence. Well-designed studies are needed to further investigate the association between anorectal disorders and SV, using standardized assessments and considering gender-specific differences among survivors.

## Data Availability

No new data were created or analyzed in this study. Data sharing is not applicable to this article.

## Appendix

Table 8.

**Table 8:** Research equations for Embase and CINAHL databases

Embase	('sexual violence'/exp OR 'sexual crime'/exp OR 'partner violence'/exp OR 'human trafficking'/exp OR 'Sexual violence*':ti,ab,kw OR 'Sexual abuse*':ti,ab,kw OR 'Sex offense*':ti,ab,kw OR 'Sexual assault*':ti,ab,kw OR 'Sexual crime*':ti,ab,kw OR 'Rape':ti,ab,kw OR 'Rapes':ti,ab,kw OR 'Genital mutilation*':ti,ab,kw OR 'female circumcision*':ti,ab,kw OR 'genital cutting*':ti,ab,kw OR 'Spouse Abuse*':ti,ab,kw OR 'partner Abuse*':ti,ab,kw OR ' Sexual Child Abuse*':ti,ab,kw OR 'Forced abortion*':ti,ab,kw OR 'Forced prostitution':ti,ab,kw OR 'sexual slavery':ti,ab,kw OR 'sexual exploitation*':ti,ab,kw OR 'human trafficking*':ti,ab,kw OR 'sex trafficking*':ti,ab,kw OR 'Sexual Harassment*':ti,ab,kw) AND ('rectum disease'/exp OR 'feces incontinence'/exp OR 'flatus incontinence'/exp OR 'defecation urgency'/exp OR 'defecation disorder'/exp OR 'painful defecation'/exp OR 'tenesmus'/exp OR 'constipation'/exp OR 'diarrhea'/de OR 'rectal disease*':ti,ab,kw OR 'rectal disorder*':ti,ab,kw OR 'rectal dysfunction*':ti,ab,kw OR 'anorectal disease*':ti,ab,kw OR 'anorectal disorder*':ti,ab,kw OR 'anorectal dysfunction*':ti,ab,kw OR 'Lower Gastrointestinal Functional':ti,ab,kw OR 'functional anorectal disease':ti,ab,kw OR 'fecal urgency':ti,ab,kw OR 'rectal urgency':ti,ab,kw OR 'fecal incontinence':ti,ab,kw OR 'anal incontinence':ti,ab,kw OR 'flatulence incontinence':ti,ab,kw OR 'flatus incontinence':ti,ab,kw OR 'passive fecal leakage*':ti,ab,kw OR 'increased daytime defecation*':ti,ab,kw OR 'nocturnal defecation*':ti,ab,kw OR 'tenesmus':ti,ab,kw OR 'constipation':ti,ab,kw OR 'feeling of incomplete bowel evacuation':ti,ab,kw OR 'straining':ti,ab,kw OR 'digitation':ti,ab,kw OR 'splinting':ti,ab,kw OR 'soiling':ti,ab,kw OR 'dyssynergic defecation*':ti,ab,kw OR 'animus':ti,ab,kw OR 'anorectal prolaps*':ti,ab,kw OR 'rectal prolaps*':ti,ab,kw OR 'anus prolaps*':ti,ab,kw OR 'rectal bleeding*':ti,ab,kw OR 'anorectal bleeding*':ti,ab,kw OR 'vaginal feces':ti,ab,kw OR 'rectovaginal fistula*':ti,ab,kw OR 'Diarrhea*':ti,ab,kw) NOT ('conference abstract'/it OR 'conference paper'/it OR 'editorial'/it OR 'letter'/it OR 'note'/it)
CINAHL	(MH "Sexual Abuse + " OR MH "Domestic Violence + " OR MH "Dating Violence" OR MH "Sexual Harassment" OR MH "Female Genital Mutilation" OR MH "Human Trafficking" OR MH "Sex Work + " OR AB ("Sexual violence*" OR "Sexual abuse*" OR "Sex offense*" OR "Sexual assault*" OR "Sexual crime*" OR "Rape" OR "Rapes" OR "Genital mutilation*" OR "female circumcision*" OR "genital cutting*" OR "Spouse Abuse*" OR "partner Abuse*" OR " Sexual Child Abuse*" OR "Forced abortion*" OR "Forced prostitution" OR "sexual slavery" OR "sexual exploitation*" OR "human trafficking*" OR "sex trafficking*" OR "Sexual Harassment*") OR TI ("Sexual violence*" OR "Sexual abuse*" OR "Sex offense*" OR "Sexual assault*" OR "Sexual crime*" OR "Rape" OR "Rapes" OR "Genital mutilation*" OR "female circumcision*" OR "genital cutting*" OR "Spouse Abuse*" OR "partner Abuse*" OR " Sexual Child Abuse*" OR "Forced abortion*" OR "Forced prostitution" OR "sexual slavery" OR "sexual exploitation*" OR "human trafficking*" OR "sex trafficking*" OR "Sexual Harassment*")) AND (MH "Rectal Diseases + " OR MH "Fecal Incontinence" OR MH "Defecation" OR MH "Constipation" OR MH "Diarrhea" OR TI ("rectal disease*" OR "rectal disorder*" OR "rectal dysfunction*" OR "anorectal disease*" OR "anorectal disorder*" OR "anorectal dysfunction*" OR "Lower Gastrointestinal Functional" OR "fecal urgency" OR "rectal urgency" OR "fecal incontinence" OR "anal incontinence" OR "flatulence incontinence" OR "flatus incontinence" OR "passive fecal leakage*" OR "increased daytime defecation*" OR "nocturnal defecation*" OR "tenesmus" OR "constipation" OR "feeling of incomplete bowel evacuation" OR "straining" OR "digitation" OR "splinting" OR "soiling" OR "dyssynergic defecation*" OR "animus" OR "anorectal prolaps*" OR "rectal prolaps*" OR "anus prolaps*" OR "rectal bleeding*" OR "anorectal bleeding*" OR "vaginal feces" OR "rectovaginal fistula*" OR "Diarrhea*") OR AB ("rectal disease*" OR "rectal disorder*" OR "rectal dysfunction*" OR "anorectal disease*" OR "anorectal disorder*" OR "anorectal dysfunction*" OR "Lower Gastrointestinal Functional" OR "fecal urgency" OR "rectal urgency" OR "fecal incontinence" OR "anal incontinence" OR "flatulence incontinence" OR "flatus incontinence" OR "passive fecal leakage*" OR "increased daytime defecation*" OR "nocturnal defecation*" OR "tenesmus" OR "constipation" OR "feeling of incomplete bowel evacuation" OR "straining" OR "digitation" OR "splinting" OR "soiling" OR "dyssynergic defecation*" OR "animus" OR "anorectal prolaps*" OR "rectal prolaps*" OR "anus prolaps*" OR "rectal bleeding*" OR "anorectal bleeding*" OR "vaginal feces" OR "rectovaginal fistula*" OR "Diarrhea*"))
